# Hypoxemic Respiratory Failure and Coccidioidomycosis-Associated Acute Respiratory Distress Syndrome

**DOI:** 10.1093/ofid/ofad679

**Published:** 2024-02-14

**Authors:** Arash Heidari, Simmer Kaur, Skyler J Pearson, Augustine Munoz, Harleen Sandhu, Gursimran Mann, Michael Schivo, Amir A Zeki, Derek J Bays, Machelle Wilson, Timothy E Albertson, Royce Johnson, George R Thompson

**Affiliations:** Department of Medicine, David Geffen School of Medicine, University of California Los Angeles, Bakersfield, California, USA; Dignity Health, Bakersfield Memorial Hospital, Bakersfield, California, USA; Valley Fever Institute, Bakersfield, California, USA; Valley Fever Institute, Bakersfield, California, USA; Division of Infectious Diseases, Department of Internal Medicine, Kern Medical, Bakersfield, California, USA; University of California–Davis Medical Center, Sacramento, CA, USA; Valley Fever Institute, Bakersfield, California, USA; Division of Pulmonary and Critical Care Medicine, Department of Internal Medicine, Kern Medical, Bakersfield, California, USA; Division of Infectious Diseases, Department of Internal Medicine, Kern Medical, Bakersfield, California, USA; University of California–Davis Medical Center, Sacramento, CA, USA; Division of Pulmonary, Critical Care, and Sleep Medicine, Department of Internal Medicine, UC Davis Lung Center, University of California Davis Medical Center, Sacramento, CA, USA; Division of Pulmonary, Critical Care, and Sleep Medicine, Department of Internal Medicine, UC Davis Lung Center, University of California Davis Medical Center, Sacramento, CA, USA; Division of Infectious Diseases, Department of Internal Medicine, University of California Davis Medical Center, Sacramento, CA, USA; Department of Public Health Sciences, University of California– Davis, Davis, California, USA; Division of Pulmonary, Critical Care, and Sleep Medicine, Department of Internal Medicine, UC Davis Lung Center, University of California Davis Medical Center, Sacramento, CA, USA; Department of Medicine, David Geffen School of Medicine, University of California Los Angeles, Bakersfield, California, USA; Valley Fever Institute, Bakersfield, California, USA; Division of Infectious Diseases, Department of Internal Medicine, Kern Medical, Bakersfield, California, USA; Division of Pulmonary, Critical Care, and Sleep Medicine, Department of Internal Medicine, UC Davis Lung Center, University of California Davis Medical Center, Sacramento, CA, USA; Department of Medical Microbiology and Immunology, University of California–Davis Medical Center, Davis, California, USA

**Keywords:** ARDS, *Coccidioides*, fungal pneumonia, glucocorticoids, steroids

## Abstract

**Background:**

Severe coccidioidomycosis presenting with respiratory failure is an uncommon manifestation of disease. Current knowledge of this condition is limited to case reports and small case series.

**Methods:**

A retrospective multicenter review of patients with coccidioidomycosis-associated acute respiratory distress syndrome (CA-ARDS) was conducted. It assessed clinical and laboratory variables at the time of presentation, reviewed the treatment course, and compared this cohort with a national database of patients with noncoccidioidomycosis ARDS. Survivors and nonsurvivors of coccidioidomycosis were also compared to determine prognostic factors.

**Results:**

In this study, CA-ARDS (n = 54) was most common in males, those of Hispanic ethnicity, and those with concurrent diabetes mellitus. As compared with the PETAL network database (Prevention and Early Treatment of Acute Lung Injury; n = 1006), patients with coccidioidomycosis were younger, had fewer comorbid conditions, and were less acidemic. The 90-day mortality was 15.4% for patients with coccidioidomycosis, as opposed to 42.6% (*P* < .0001) for patients with noncoccidioidomycosis ARDS. Patients with coccidioidomycosis who died, as compared with those who survived, were older, had higher APACHE II scores (Acute Physiology and Chronic Health Evaluation), and did not receive corticosteroid therapy.

**Conclusions:**

CA-ARDS is an uncommon but morbid manifestation of infection. When compared with a national database, the overall mortality appears favorable vs other causes of ARDS. Patients with CA-ARDS had a low overall mortality but required prolonged antifungal therapy. The utility of corticosteroids in this condition remains unconfirmed.

Coccidioidomycosis is an invasive fungal infection caused by the dimorphic fungi *Coccidioides immitis* and *C posadasii.* Upon inhalation of the arthroconidia (eg, spores), 60% of those exposed develop asymptomatic infection or a mild respiratory illness [1, 2]. The remainder develop a myriad of symptoms ranging from fever, chills, arthralgias (“valley fever”), and pneumonia to severe and life-threatening infections [3].

Acute respiratory distress syndrome (ARDS) is an inflammatory pulmonary condition that often leads to hypoxic respiratory failure and is associated with an all-cause mortality of up to 40% [4]. Although ARDS is most commonly associated with sepsis and pneumonia [5], it is an uncommon manifestation of pulmonary coccidioidomycosis, with only case reports and small case series available in the literature [6]. When ARDS develops in the setting of coccidioidomycosis infection, it is typically the presenting manifestation of those with previously undiagnosed coccidioidomycosis [7]. The medical community's understanding of coccidioidomycosis-associated ARDS (CA-ARDS) is incomplete and has been based solely on case reports. Prior publications have suggested a high inoculum and/or host immunosuppression as significant risk factors, and this syndrome has an observed mortality of 35% to 50% [8, 9].

The treatment of ARDS has significantly advanced in recent years, and glucocorticoid therapy has been advocated for some patient groups. The clinical profiles of adult patients with documented coccidioidomycosis and ARDS and the potential role of adjunctive glucocorticoid therapy are described. The cohort of CA-ARDS is additionally compared with an established national database of patients with ARDS attributed to noncoccidioidomycosis causes (PETAL Network; Prevention and Early Treatment of Acute Lung Injury).

## METHODS

A multicenter collaborative group performed a pooled retrospective analysis of all patients with coccidioidomycosis-associated respiratory failure (Kern Medical, Bakersfield, CA; UC Davis Medical Center, Sacramento, CA). Cases were identified by searching hospital databases by *ICD-9* and *ICD-10* codes. Abstracted data included demographic and clinical information, results of laboratory and radiographic studies, outcomes, and treatment regimens, as well as microbiology, pathology, and serologic results. Patients with ARDS caused by coccidioidomycosis between 2009 and 2019 were included in the analysis.

Inclusion criteria comprised a proven or probable diagnosis of coccidioidomycosis by revised criteria from the Mycoses Study Group and European Organization for Research and Treatment of Cancer [10]. ARDS was defined by the Berlin definition: acute onset of respiratory symptoms beginning with a known insult, bilateral opacities on chest radiography, respiratory failure not explained by cardiac failure or fluid overload, and moderate to severe impairment of oxygenation (defined as a PaO_2_:FiO_2_ ratio <300) [11]. Patients with coinfections or alternative diagnoses (eg, noncoccidioidomycosis) were excluded from the analysis. Patients were considered survivors if they were discharged from the hospital in stable condition without plans for palliative or hospice care.

Patients in the cohort with coccidioidomycosis-associated ARDS were matched ∼1:20 with patients in the PETAL Network database who did not have fungal disease as the etiology of ARDS [12]. PETAL is a multicenter clinical trials network funded by the National Heart, Lung, and Blood Institute (National Institutes of Health) to conduct clinical trials to prevent and treat ARDS.

Chi-square, Fisher exact, and Wilcoxon rank sum tests were used to compare groups. All statistical analyses were conducted with SAS software version 9.4 for Windows (SAS Institute Inc).

### Patient Consent Statement

The UC Davis Medical Center and Kern Medical institutional review boards approved this study with a waiver of patient consent, given the retrospective nature of data collection.

## RESULTS

Fifty-four patients with coccidioidomycosis and hypoxic respiratory failure due to ARDS met inclusion and exclusion criteria (Table 1). Patients’ median age was 42.5 years (range, 18–85). The majority of study participants were male (75.9%) and Hispanic (66.7%). The most frequent comorbid conditions were diabetes (44.4%), hypertension (27.8%), cirrhosis (7.4%), heart disease (5.6%), and HIV (1.9%). Most patients had nondisseminated forms of coccidioidomycosis with primary pulmonary disease as the most common form (85.9%), followed by cavitary pneumonia (8.5%) and disseminated infection (5.6%). Presenting clinical manifestations were nonspecific and consistent with prior reports of coccidioidomycosis [13]: cough (88.9%), fever (81.5%), chills (59.3%), chest pain (57.4%), night sweats (44.4%), fatigue (27.8%), headache (22.2%), myalgia (20.4%), and weight loss (16.7%).

**Table 1.** Demographic Characteristics of Patients With Coccidioidomycosis-Associated ARDS (n = 54)

	Median (IQR) or No. (%)
Age, y	42.5 (32.3–50.8)
Male sex	41 (75.9)
Race/ethnicity	
Hispanic/Latino	36 (66.7)
Caucasian	10 (18.5)
African American	7 (13.0)
Oceanic/Filipino	1 (1.9)
Presenting symptom	
Cough	48 (88.9)
Fever	44 (81.5)
Chills	32 (59.3)
Chest pain	31 (57.4)
Night sweats	24 (44.4)
Fatigue	15 (27.8)
Headache	12 (22.2)
Myalgia	11 (20.4)
Weight loss	9 (16.7)
Arthralgia	3 (5.6)
Rash	3 (5.6)

Abbreviation: ARDS, acute respiratory distress syndrome.

Laboratory values on admission (Table 2) included a median white blood cell count of 15.4 cells/μL and a median eosinophil count of 0.4 cells/μL. The median PaO_2_/FiO_2_ ratio was 247 (range, 63–300). Coccidioidal serologies were positive in all patients in this cohort, with IgM positivity noted in 46 (64.8%) and IgG positivity noted in 44 (62.0%; 19 were positive for IgM and IgG anticoccidioidal antibodies). The median coccidioidomycosis complement fixation antibody titer was 1:2 (range, <1:2–1:128). Blood cultures were positive for *Coccidioides* spp in only 1 patient.

**Table 2.** Presenting Laboratory and Radiographic Manifestations in Patients With Coccidioidomycosis-Associated ARDS (n = 54)

	Median (Range) or No. (%)
Total white blood cells, cells/μL	15.4 (4.4–31.6)
Absolute eosinophils, cells/μL	0.4 (0–3.66)
PaO_2_/FiO_2_ ratio	247 (63–300)
Coccidioidal serologies^a^	
IgM positive	46 (64.8)
IgG positive	44 (62.0)
Complement fixation titer	1:2 (<1:2–1:128)
Blood cultures positive for *Coccidioides*	1 (1.4)
Radiographic presentation^b^	
Alveolar	43 (60.6)
Nodular	12 (16.9)
Mixed alveolar-nodular	10 (14.1)
Miliary	6 (8.5)
Cavity	6 (8.5)
Mediastinal or hilar lymphadenopathy	11 (20.4)
Admission bronchoscopy	23 (42.6)

Abbreviation: ARDS, acute respiratory distress syndrome.

^a^Patients could be positive for IgM and IgG anticoccidioidal antibodies.

^b^Patients with cavitary lesions all had additional radiographic manifestations.

Radiographic patterns on admission included several manifestations, such as alveolar (60.6%), nodular (16.9%), and mixed alveolar-nodular (14.1%). Miliary disease was seen in only 8.5% of patients. Patients with cavities (8.5%) all had additional radiographic manifestations. Hilar and/or mediastinal lymphadenopathy was present in 20.4% of patients. Bronchoscopy was used in 42.6% in an attempt at early diagnosis. In this group, 15 of 23 (65.2%) had a diagnosis made by bronchoscopic bronchoalveolar lavage with either cytology or culture.

Treatment consisted primarily of regimens containing an amphotericin B formulation (98.1%). Amphotericin B alone was used in 76.1% (lipsomal amphotericin B, 48.1%; amphotericin B lipid complex, 27.8%). Combination therapy with an amphotericin B formulation and a triazole was used in 25.9%. Fluconazole was monotherapy in a minority of patients (6.5%). Adjunctive corticosteroid therapy was given to 25.9%, with the majority receiving a prednisone taper analgous to that used in the treatment of *Pneumocystis* pneumonia [14], while 6 patients received variable amounts of methyprednisolone (0.5–1 mg/kg for 5–7 days).

A comparison of demographic variables and mortality between CA-ARDS and patients in the PETAL database is outlined in Table 3. Those in the nationwide PETAL database were older (*P* < .0001), less commonly male (*P* = .0034), and more likely to be Caucasian and less likely to be of Hispanic ethnicity (*P* < .0001). Patients with CA-ARDS were also more likely to have diabetes mellitus but less likely to have hypertension, use tobacco, or have underlying chronic obstructive pulmonary disease or pulmonary disease.

**Table 3.** Differences Among Patients With Coccidioidomycosis-Associated ARDS and Non–Coccidioidomycosis-Associated ARDS: PETAL Database

	Coccidioidomycosis-Associated ARDS, Median (IQR) or No. (%)		
	Yes (n = 54)	No (n = 1006)	*P* Value
Age, y	42.5 (32–51)	58 (46–66)	<.0001
Male sex	41 (75.9)	560 (55.7)	.0034
Ethnicity			
Hispanic/Latino	36 (66.7)	118 (11.7)	<.0001
Caucasian	10 (18.5)	703 (69.9)	
African American	7 (13.0)	141 (14.0)	
Other	1 (1.9)	30 (3.0)	
Comorbid condition			
Diabetes mellitus	24 (44.4)	301/967 (31.1)	.0409
Hypertension	15 (27.8)	499/967 (51.6)	.0007
Tobacco	8 (14.8)	280/548 (51.1)	<.001
Cirrhosis	4 (7.4)	101/968 (10.4)	.4759
Hepatic failure	0	46/968 (4.8)	
CAD/CHF	3 (5.6)	82/967 (8.5)	.4491
HIV/AIDS	1 (1.9)	17/969 (1.8)	.9577
COPD/pulmonary disease	1 (1.9)	186/968 (19.2)	.0013
Leukemia/lymphoma	0	58/968 (6.0)	.0675
Malignancy with metastases	0	52/968 (5.4)	.1059
Laboratory			
pH	7.4 (7.4–7.5)	7.3 (7.3–7.4)	<.0001
PaCO_2_	36 (32–40)	42 (37–49)	<.0001
PaO_2_	64 (55.5–74.5)	76 (67–92)	.8271
PaO_2_/FiO_2_ ratio	247.8 (171.6–292.7)	110 (85–136)	<.0001
Adjunctive measure			
Corticosteroids	14 (25.9)	387/1006 (38.5)	.0641
Vasopressors	0	795/1006 (79.0)	<.0001
ECMO	0	13/1006 (1.3)	>.99
Mortality at 90 d	8 (14.8)	429 (42.6)	<.0001

Abbreviations: ARDS, acute respiratory distress syndrome; CAD, coronary artery disease; CHF, congestive heart failure; COPD, chronic obstructive pulmonary disease; ECMO, extracorporeal membrane oxygenation; PETAL, Prevention and Early Treatment of Acute Lung Injury Clinical Trials Network of the National Heart, Lung, and Blood Institute.

Laboratory values on admission exhibited signficant differences. Noncoccidioidomycosis cases had lower blood pH, higher PaCO_2_, and lower PaO_2_/FiO_2_ ratios on admission (*P* < .05). PaO_2_ values were lower in the CA-ARDS group vs the noncoccidioidomycosis group (median, 64 vs 76 mm Hg) but not statistically significant (*P* = .83). Despite these lower PaO_2_ values, the overall 90-day mortality was significantly lower in patients with CA-ARDS (15.4%) than those with ARDS associated with other causes (42.6%, *P* < .0001). Patients in the PETAL database received significantly more vasopressors (*P* < .0001), and several received extracorporeal membrane oxygenation (1.3%), suggesting that this group had a higher demand for health care resources due to severity of illness.

In the comparison between those who survived CA-ARDS and those who did not (Table 4), there were no statistically significant differences in age, sex, ethnicity, or comorbidities with the exception of underyling cirrhosis (*P* = .008), which appeared to be a signifcant risk factor for death. The type of infection and laboratory parameters were not significantly different between groups, although the PaO_2_/FiO_2_ ratio was numerically lower in the group that did not survive, as would be expected. APACHE II (Acute Physiology and Chronic Health Evaluation) scores were also lower in survivors (*P* = .0335).

**Table 4.** Analysis of Patients With Coccidioidomycosis-Associated ARDS Who Survived vs Died

	Median (IQR) or No. (%)		
	Survived (n = 46)	Died (n = 8)	*P* Value
Age, y	42 (29–49.8)	47 (39.8–55)	.0824
Male sex	35 (76)	6 (75)	.9471
Race/ethnicity			
Hispanic/Latino	31 (67.4)	5 (62.5)	.7865
Caucasian	8 (17.4)	2 (25)	.6091
African American	6 (13.0)	1 (12.5)	.5975
Other	1 (2.1)	0	
Comorbid condition			
Diabetes mellitus	21 (45.7)	3 (37.5)	.7201
Hypertension	10 (21.7)	5 (62.5)	.0514
Tobacco	5 (10.9)	3 (37.5)	.0856
Cirrhosis	1 (2.2)	3 (37.5)	.0084
CAD/CHF	2 (4.3)	1 (12.5)	.388
HIV/AIDS	1 (2.2)	0	>.99
COPD/pulmonary disease	1 (2.2)	0	>.99
Laboratory values			
pH	7.4 (7.4–7.5)	7.4 (7.3–7.5)	.3051
PaCO_2_	36 (32–40)	33.5 (31.5–40.5)	.8720
PaO_2_	64 (55–70)	78.5 (60–103)	.8782
PaO_2_/FiO_2_ ratio	252.8 (188.6–296.4)	184.6 (102.3–283.2)	.3219
APACHE II	8 (3–11)	14 (9.5–19)	.0335
Type of coccidioidal infection: dissemination	4	0	>.99
Total white blood cells, cells/μL^a^	16.09 (4.6–26.3)	13.8 (4.4–25.8)	.56
Absolute eosinophils, cells/μL^a^	0.68 (0–2.84)	0.97 (0–3.66)	.28
CF titer^a^	1:19 (0–1:128)	1:18 (0–1:64)	.70
Blood cultures (+) for *Coccidioides*	0	1	.15
Presentation: miliary disease	4 (8.7)	2 (25)	.2127
Initial antifungal therapy			
Amphotericin B	45 (97.8)	8 (100)	>.99
Fluconazole	3 (6.5)	0	>.99
Combination^b^	14 (30.4)	0	.0953
Corticosteroids	10 (21.7)	4 (50)	.1832
*Pneumocystis* protocol^c^	8 (17.4)	0	.546
Hospitalization, d^a^	53.9 (6–208)	17.4 (2–60)	.003

Abbreviations: APACHE II, Acute Physiology and Chronic Health Evaluation; ARDS, acute respiratory distress syndrome; CAD, coronary artery disease; CF, complement fixation; CHF, congestive heart failure; COPD, chronic obstructive pulmonary disease.

^a^Mean (range).

^b^Patients could receive more than one agent simultaneously.

^c^See reference [14].

Patients with CA-ARDS who were treated with corticosteroids were more likely to survive than those who did not receive steroids, but this did not reach statistical signifcance (*P* = .06). The comparison is likely underpowered in this analysis given the relative rarity of this complication from coccidioidomycosis treated with steroids.

## DISCUSSION

Severe presentations of primary coccidioidomycosis are uncommon and are typically observed in patients with underlying immunologic deficits or following a high burden of exposure resulting in diffuse multilobar pneumonia. Respiratory failure and ARDS are primarily observed within these groups [15].

The acute, diffuse, inflammatory lung injury observed in ARDS is difficult to treat and requires a multidisciplinary approach to enable a timely diagnosis and for appropriate treatment to be initiated in an expedited fashion [16]. In this cohort, the majority of patients were middle-aged men, consistent with patient sex and epidemiology of hospitalized coccidioidomycosis infections observed in other series [17, 18].

This cohort had a high prevalence of diabetes mellitus, as seen in case series of ARDS caused by other fungal pathogens [19]. In contrast, a recently conducted systematic review suggests that underlying diabetes does not predispose or increase the mortality of ARDS, although few fungal cases were included in these studies [20].

We did observe several other underlying causes of immunosuppression in our patients. HIV/AIDS, cirrhosis, chronic kidney disease, and chronic obstructive pulmonary disease accounted for close to a quarter of all patients. Patients with these underlying immunologic and/or structural lung defects accounted for a large percentage of all ARDS cases, similar to other reports [21]. These patients may experience a higher burden of infection or potentially develop symptoms after a greater duration of illness, resulting in delayed diagnosis and treatment. It is of interest that cirrhosis appeared to be the major risk factor for death in our study. Chronic liver disease is a known immunocompromising illness that has been associated with mortality in other fungal diseases (*Candida* [22] and *Cryptococcus* [23]) but not definitely demonstrated in coccidioidomycosis [24].

The majority of patients presenting with CA-ARDS in this series had primary pulmonary infection (85.9%). Previous reports indicate that patients with ARDS likely had a high burden of *Coccidioides* exposure, although we are unable to verify this association in our retrospective series, which lacks detailed exposure information [25]. Prior reports also noted an association between miliary disease and ARDS [15, 26]; however, we found that this radiographic manifestation was the least common radiographic presentation. Diffuse pneumonia with reticular, nodular, or reticulonodular features was the most frequently encountered lung radiographic pattern. The serologic titers observed in this study were relatively low. CA-ARDS is a severe manifestation of acute coccidioidomycosis, and complement fixation titers (IgG) did not likely increase to their maximum titer. Antifungal therapy may abrogate the development of IgG antibody production and/or blunt the serologic increase observed with ongoing fungal disease, and any positive serologic result in a patient with a potential presentation of coccidioidomycosis should not be ignored [27–29].

The treatment of ARDS is largely supportive, aimed at preventing further lung injury with rapid treatment of the underlying cause—all paramount for a successful outcome. Only 2 management strategies are known to improve mortality in ARDS: (1) the ARDSNet's low tidal volume strategy during mechanical ventilation and (2) prone positioning for those with more severe hypoxemic forms of ARDS [30]. Unfortunately, these events were not captured in the coccidioidomycosis database.

In ARDS cases secondary to coccidioidomycosis infection, aggressive antifungal therapy is indicated, as observed in the large proportion of patients in this series treated with an amphotericin B formulation. There are no comparative trials of polyenes vs triazoles, although polyene therapy has been uniformly recommended in severe cases [1]. Combination therapy is attractive, as it may offer more rapid fungicidal activity [31]. We observed the use of this approach in 25.9% of our study participants, although data in this regard are scarce and the theoretical benefits are largely extrapolated from other fungal infections and murine models of infection [31], with no clear benefit seen in our cohort.

The use of systemic glucocorticoid therapy in patients with ARDS remains controversial and is dependent on the precipitating cause, associated comorbidities, and severity of illness. The use of systemic corticosteroid treatment with ARDS also depends in part on regional institutional practices. In patients with fungal-associated ARDS, there is a dearth of data, and it is unlikely that a prospective controlled trial can be feasibly conducted to address this question. ARDS secondary to blastomycosis, histoplasmosis, and paracoccidioidomycosis has been described; however, the utility of corticosteroid therapy in these conditions similarly remains unconfirmed [19, 32–38]. Concerns for promoting fungal dissemination during glucocorticoid therapy have been posed, yet in the setting of concurrent antifungal therapy, this was not observed in any patient. Conversely, patients with pneumonia and impaired oxygenation caused by the fungus *Pneumocystis jirovecii* have been shown to significantly benefit from corticosteroid therapy in the HIV/AIDS population [39].

In contrast to these other causes of fungal pneumonia, coccidioidomycosis often manifests with significant eosinophilia evident on peripheral blood and bronchoalveolar lavage samples [26, 40]. Thus, the anti-inflammatory and eosinopenic effects of corticosteroids could theoretically lead to clinical benefit, particularly if a larger data set becomes available. The duration of symptom onset to time to receipt of corticosteroids and/or antifungals is also a potential confounder, but symptom duration was not available in this retrospective report. Physician-specific differences were seen in our review, with some practitioners preferring a steroid taper as used in *P jirovecii* pneumonia (prednisone, 40 mg, twice daily × 5 days, followed by 40 mg daily for 5 days, followed by 20 mg daily for 11 days) in combination with antifungal therapy, while other sites preferred antifungal therapy alone. It did not appear that the presence of blood or lung eosinophilia affected the clinical decision to use corticosteroids in CA-ARDS. This remains an open question for future clinical investigation.

The retrospective nature of this report has several important limitations. Inherent patient-specific differences may have led clinicians to opt for treatment regimens and decision processes that are not adequately captured on our chart review. For example, somewhat paradoxically, patients treated with corticosteroid therapy had a numerically higher P_a_O_2_/FiO_2_ ratio. Perhaps these patients were thought to have a more favorable prognosis and thus adjunctive therapy was offered, as compared with those not offered corticosteroids, where treatment may have been considered futile by clinicians caring for the patients, although this remains speculative. Patients with less severe disease (eg, PaO_2_/FiO_2_ ratios <350) also may benefit from corticosteroids, but these patients were excluded from inclusion given the criteria used in the PETAL trial.

This study compared a cohort with coccidioidomycosis-associated ARDS with one of the large PETAL trials to explore differences between fungal-associated ARDS and a larger national database to evaluate key presenting and prognostic differences. Overall patients with coccidioidomycosis were considerably younger; were more likely to be male, Hispanic, or other ethnicity; and have diabetes mellitus. The PETAL database cohort had more comorbidities—particularly, hypertension, tobacco use, and chronic obstructive pulmonary disease—and lower arterial pH values, presumably as ARDS often accompanied septic shock. The mortality rates were significantly different between groups, potentially also associated with the higher age, comorbidity rate, and later presentation in PETAL.

One possible confounding variable is the fact that ARDS itself can manifest as 2 recently described phenotypes, including a hypo- vs hyperinflammatory type [41, 42]. Whether CA-ARDS fits either of these subphenotypes is not yet clear. However, future research in this area must consider this divergence in ARDS clinical phenotypes, and this could shed much-needed light on this *Coccidioides*-associated syndrome, particularly since there was a trend to improved mortality with adjunctive glucocorticoid treatment in CA-ARDS.

ARDS due to coccidioidomycosis infection remains a severe but uncommon manifestation of this infection and carries a relatively high morbidity and mortality rate, although the prognosis appears favorable when compared with a national database of patients with ARDS. Advances in the treatment of these patients are urgently needed, and in the absence of prospective clinical trials, case series and consensus opinion are likely to guide therapy. The current report is the largest patient series of CA-ARDS to date, and we describe our combined experience across multiple sites.

Attempts to ascertain an early diagnosis, instillation of aggressive antifungal therapy, and consideration of corticosteroid therapy should be pursued in selected patients. In addition, identification of patients with cirrhosis, coccidiomycosis, and ARDS may be important in clarifying prognosis, treatment options, and goals of care.

## Notes

***Financial support*.** This work was supported by the National Center for Advancing Translational Sciences, National Institutes of Health (UL1 TR001860); the National Institutes of Health (5U19AI166798-02); and the UC-Davis Burden Family Gift Fund for research on coccidioidomycosis.
